# Allergens of the entomopathogenic fungus *Beauveria bassiana*

**DOI:** 10.1186/1476-7961-3-1

**Published:** 2005-01-11

**Authors:** Greg S Westwood, Shih-Wen Huang, Nemat O Keyhani

**Affiliations:** 1 Department of Microbiology and Cell Science, University of Florida, Gainesville, FL 32611, USA; 2 Department of Pediatrics, University of Florida, College of Medicine, 32610, USA

## Abstract

**Background:**

*Beauveria bassiana *is an important entomopathogenic fungus currently under development as a bio-control agent for a variety of insect pests. Although reported to be non-toxic to vertebrates, the potential allergenicity of *Beauveria *species has not been widely studied.

**Methods:**

IgE-reactivity studies were performed using sera from patients displaying mould hypersensitivity by immunoblot and immunoblot inhibition. Skin reactivity to *B. bassiana *extracts was measured using intradermal skin testing.

**Results:**

Immunoblots of fungal extracts with pooled as well as individual sera showed a distribution of IgE reactive proteins present in *B. bassiana *crude extracts. Proteinase K digestion of extracts resulted in loss of IgE reactive epitopes, whereas EndoH and PNGaseF (glycosidase) treatments resulted in minor changes in IgE reactive banding patterns as determined by Western blots. Immunoblot inhibitions experiments showed complete loss of IgE-binding using self protein, and partial inhibition using extracts from common allergenic fungi including; *Alternaria alternata*, *Aspergillus fumigatus*, *Cladosporium herbarum*, *Candida albicans*, *Epicoccum purpurascens*, and *Penicillium notatum*. Several proteins including a strongly reactive band with an approximate molecular mass of 35 kDa was uninhibited by any of the tested extracts, and may represent *B. bassiana *specific allergens. Intradermal skin testing confirmed the *in vitro *results, demonstrating allergenic reactions in a number of individuals, including those who have had occupational exposure to *B. bassiana*.

**Conclusions:**

*Beauveria bassiana *possesses numerous IgE reactive proteins, some of which are cross-reactive among allergens from other fungi. A strongly reactive potential *B. bassiana *specific allergen (35 kDa) was identified. Intradermal skin testing confirmed the allergenic potential of *B. bassiana*.

## Background

Microorganisms are currently under intensive study for use as biopesticides [1-3]. Several fungal species including *Metarhizium anisopliae*, *Verticillium lecanii*, and *Beauveria bassiana *are being used as biocontrol agents for a number of crop, livestock, and human nuisance pests [4-7]. Strains of *B. bassiana *have been licensed for commercial use against whiteflies, aphids, thrips, and numerous other insect and arthropod pests. *B. bassiana *fungal formulations are being spread onto a range of vegetables, melons, tree fruits and nuts, as well as organic crops. As alternatives to chemical pesticides these agents are natural occurring and are considered to be non-pathogenic to humans, although a few cases of *B. bassiana *mediated tissue infections have been reported [8,9].

Airborne mold spores are widespread, and many have been identified as inhalant allergens eliciting type I hypersensitive reactions in atopic individuals [10-14]. Common allergenic moulds include the anamorphs of ascomycetes and constitute many species within the *Alternaria*, *Aspergillus*, and *Cladosporium *genera [15-19]. The genes encoding for numerous fungal allergens have been isolated, and their protein products expressed and characterized. Purified fungal allergens have been shown to be bound by human IgEs and to elicit allergic reactions in atopic individuals using skin prick tests. Patients with mould allergies often display IgE-mediated responses to multiple fungi, a phenomenon typically thought to result from the presence of common cross-reactive allergen(s) [15,20-22], although parallel independent sensitization to multiple fungal allergens can also occur. In this regards, identification of genus and/or species specific allergens would provide useful tools in differentiating allergic reactions due to primary sensitization and those mediated by cross-reactive epitopes.

In the present study, we demonstrate *Beauveria bassiana *crude extracts contain numerous allergens capable of being recognized by human serum IgEs. The allergens were proteinaceous in nature, and immunoblot inhibition experiments revealed the presence of shared epitopes between *Beauveria *and several other common fungal moulds. Potential *Beauveria-specific *allergens were also identified, including a strongly reactive ~35-kDa protein band. Intradermal skin testing using *B. bassiana *extracts resulted in allergenic reactions in several individuals, including some who have had occupational exposure to the fungus.

## Methods

### Strains and cultures

*Beauveria bassiana *(ATCC 90517) was grown on Sabouraud dextrose + 0.5–1% yeast extract or Potato dextrose (PD) media on either agar plates or in liquid broth. Plates were incubated at 26°C for 10–12 days and conidia were harvested by flooding the plate with sterile dH_2_O containing 0.01% Tween-20. Liquid cultures were inoculated with conidia harvested from plates at 0.5–1 × 10^5 ^conidia/ml.

### Extract preparation

*Alternaria alternata*, *Aspergillus fumigatus*, *Candida albicans*, *Cladosporium herbarum*, *Epicoccum purpurascens*, and *Penicillium notatum *were acquired from Greer Laboratories inc., (Lenoir, NC). Extracts were resuspended in TE (40 mM Tris-HCl, pH 8.0, 1 mM EDTA) to a final concentration of 2 mg/ml. *Beauveria bassiana *was grown in Sabouraud's broth containing 1% yeast extract with aeration at 25°C for 3–5 d. Cellular mass was harvested by centrifugation (10,000 × g, 10 min) and freeze-dried. Cells were resuspended in TE containing 0.1% phenylmethylsulfonyl fluoride (PMSF) and homogenized using a bead-beater apparatus.

### Precipitations

Crude extracts of *B. bassiana *were subjected to three successive precipitations before use in Western blots.

#### Acetone precipitation

Homogenized *B. bassiana *extracts (50 ml) were mixed with 8 × volume (400 ml) of acetone (kept at -20°C), with rapid stirring, and incubated overnight at -20°C. The precipitate was collected by centrifugation (30 min, 4000 × g), and the pellet was air dried (10 min) before being resuspended in TE containing 0.1% PMSF.

#### Streptomycin precipitation (removal of DNA)

Streptomycin sulfate (5 ml of 10% solution) was added dropwise to resuspended acetone precipitated extracts (40 ml) at 4°C with rapid stirring. Samples were incubated for an additional 30 min on ice before being centrifuged (15 min, 10,000 × g) in order to remove the precipitate. Proteins in the resultant supernatant were precipitated using ammonium sulfate.

#### Ammonium sulfate

The proteins present in the streptomycin sulfate treated supernatant were precipitated using ammonium sulfate (75%, final concentration). Saturated ammonium sulfate (120 ml) was added dropwise to the *Beauveria *extract (40 ml) at 4°C with rapid stirring. The solution was allowed to stir overnight at 4°C and precipitated proteins were harvested by centrifugation (30 min, 100,000 × g). The protein pellet was resuspended in TE containing 0.1% PMSF (40 ml) and extensively dialyzed against the same buffer before use.

#### SDS-Polyacrylamide gel electrophoresis (PAGE)

Protein samples (30–40 μg) were analyzed by sodium-dodecyl-sulphate-polyacrylaminde gel electrophoresis (SDS-PAGE, 10% Bis-tris gel, Invitrogen, Carlsbad, CA) using standard protocols. Gels were stained with Gelcode blue stain reagent (Pierce, Rockford, IL) and subsequently de-stained with dH20.

### Western blotting

Protein samples were separated under reducing conditions using 10% Bis-tris polyacrylamide gels (Invitrogen Mops system) and transferred to polyvinylidene-fluoride (PVDF) membranes (Invitrogen) as described. Immunoblot experiments were performed using individual and pooled human sera as the primary antibody solution as indicated. Typically, sera were diluted 1:5 with Tris-HCl buffered saline (TBS) containing 5% dry milk + 0.1% Tween-20. IgE-specific reactivity was visualized using a horseradish peroxidase (HRP) conjugated goat anti-human IgE (polyclonal) secondary antibody (BioSource International, Los Angeles, CA). Membranes were washed with TBS containing 0.1% Tween-20 and bands were visualized using the Immuno-Star HRP detection system (Biorad, Hercules, CA).

### Enzyme Treatments

The ammonium sulfate fraction of *B. bassiana *crude extracts was treated with Proteinase K (ICN-Biomed, Aurora, Oh) following standard protocols. Typically, samples (36 μl) were incubated with 4 μl Proteinase K solution (10 mg/ml in 50 mM Tris-HCl, pH 7.5) for 2 hr at 37°C before analysis. Samples were also treated with endoglycosidase-H (EndoH, New England Biolabs, Beverly, MA) and peptide: N-Glycosidase F (PNGaseF, New England Biolabs) according to the manufacturer's recommendations. For EndoH and PNGaseF treatments, samples (36 μl) were denatured in 4 μl 10 × denaturing buffer (0.5% SDS, 1% β-mercaptoethanol) at 100°C for 10 min prior to the addition of the EndoH (5 μl of 10 × G5 Reaction Buffer, 50 mM sodium citrate, pH 5.5) and PNGaseF reaction buffers (50 mM sodium phosphate pH 7.5) and enzymes (5 μl), respectively. Reactions were incubated at 37°C for 2 h before being analyzed by SDS-PAGE and Western blotting.

### Immunoblot inhibition

IgE binding to *B. bassiana *proteins were competed with proteins of other fungal extracts. SDS-PAGE resolved *B. bassiana *proteins were electroblotted to PVDF membranes as described above. Membranes were blocked with TBS containing 5% dry milk + 0.1% Tween-20 and strips were incubated with pooled human sera (1:5 v/v in same buffer) containing 100–500 μg of the indicated fungal crude protein extract.

### Skin sensitivity profiles to fungal extracts

Patients were tested with 9 common fungal extracts for allergy diagnosis using a skin prick assay. The following extracts were obtained from ALA-Abello (Round Rock, TX); *Alternaria tenius*, *Aspergillus fumigatus*, *Cephalosporium *(*Acremonium strictum*), *Curvularia *spp. *Bipolaris*, *Epicoccum nigram*, *Fusarium *spp., *Helminthosporium sativum*, *Hormodendrum horde*, *Penicillium *(mixed, *P. chrysogenum *and *P. notatum*). Extracts were tested using a 1:10 dilution of the 20,000 PNU/ml stock solution, and skin sensitivity was recorded on a relative scale from 0–4 reflecting the size of induration or weal (4 representing the highest reactivity) and using histamine (0.1 mg/ml) reaction scored as a 3 if no interference was present.

### Intradermal skin testing

*B. bassiana *crude extracts were prepared as described above but were extensively dialyzed against 0.15 N NaCl and filtered through a 0.22 μm filter before use. Subjects were given intradermal injections of 0.1 ml crude extract ranging in concentration from 0.01–1 mg/ml. Control injections included saline and histamine (0.1 mg/ml). Allergenic reactions were allowed to develop for 15–30 min before the height and width of the reactions were recorded.

## Results

### Identification of IgE reactive bands

**Figure 1:**
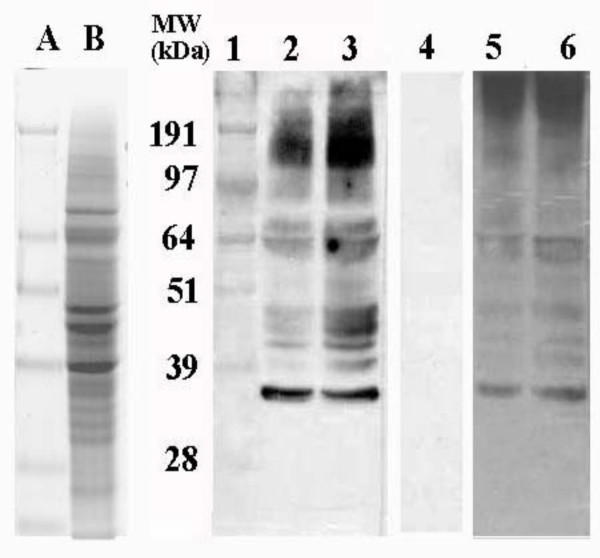
SDS-PAGE and immunoblot analysis of *Beauveria bassiana *crude extracts. SDS-PAGE, Gelcode blue stained, lanes A) 5 μg protein standards, and B) 40 μg *B. bassiana *crude extract. Immunoblots probed with pooled serum mix-I (patients displaying mould allergies), lanes 1), 5 μg protein standards, 2) 20 μg *B. bassiana *crude extract, 3) 40 μg crude extract, 4) 40 μg crude extract, Proteinase K treated, 5) 40 μg crude extract, denaturing buffer control (no EndoH), 6) 40 μg crude extract, EndoH treated

An ammonium sulfate fraction of *B. bassiana *proteins was resolved on SDS-PAGE (Fig. 1, lane B) and transferred to PVDF membranes as described in the Materials and Methods. Membranes were probed with sera from individual patients who were reactive to various moulds (Table 1), which was pooled and used to demonstrate IgE reactivity against antigens present in *B. bassiana *extracts (Fig. 1). Serum mix-I represents pooled sera derived from patients E, J, K, L, and M, as well as three additional patients that were only tested (skin prick) against *Aspergillus *and *Penicillium*, displaying test scores of 3–4 for each. These data demonstrate human IgE binding of allergens present in *B. bassiana *extracts. Initial blots showed 12–15 distinct reactive protein bands, ranging in molecular mass from 12 kDa to >95 kDa (under denaturing conditions); with the most prominent bands located around 64, 45, and 35 kDa. Control experiments omitting either the primary or secondary antibody incubation steps resulted in complete loss of signal. Proteinase K digestion of samples also resulted in loss of all signal (Fig. 1, lane 4), indicating the proteinaceous nature of the IgE reactive bands. Since the carbohydrate moieties of several protein allergens are known to play a role in their allergenicity and even cross-reactivity [20-22], samples were treated with the deglyocosylating enzymes EndoH and PNGaseF. Control experiments incubating samples in the EndoH denaturing buffer without any enzyme altered the IgE-reactive signals (Fig. 1, lane 5), however, samples treated with EndoH did not appear any different than control reactions (Fig. 1, lane 6). Similar results were obtained in PNGaseF digests (data not shown). These data appear to indicate that the *B. bassiana *IgE-antigen profiles observed on Western blots are proteins with minimal contributions due to glycosylation.

**Table 1:** Allergic profile of patients A–G, obtained by skin prick testing.

Patient ID	Individual Skin Reactivity to Fungal Extracts*								
	
	*Alt*^†^	*Asp*	*Cep*	*Cur*	*Epi*	*Fusa*	*Helmin*	*Hormo*	*Pen*
A	3	2	0	3	2	2	0	0	3
B	3	2	0	2	3	2	2	2	0
C	4	0	0	3	0	2	0	0	0
D	0	0	2	2	0	2	0	2	2
E	3	2	0	3	2	3	3	0	0
F	4	1	1	2	4	0	2	0	0
G	3	0	0	4	3	0	2	0	0

*Skin test scores were registered using a relative scale from 0–4 with 4 representing the highest reactivity as described in the Materials and Methods. ^†^Abbreviations used; *Alt*, *Alternaria tenius*; *Asp*, *Aspergillus fumigatus*; *Cep*, *Cephalosporium *(*Acremonium strictum*); *Cur*, *Curvularia *spp. *Bipolaris*; *Epi*, *Epicoccum nigram*; *Fusa*, *Fusarium *spp.; *Helmin*, *Helminthosporium sativum*; *Hormo*, *Hormodendrum horde*; *Pen*, *Penicillium *(mixed, *P. chrysogenum *and *P. notatum*).

### Immunoprint Analysis of *B. bassiana*: Reactivity with Individual Sera

**Figure 2:**
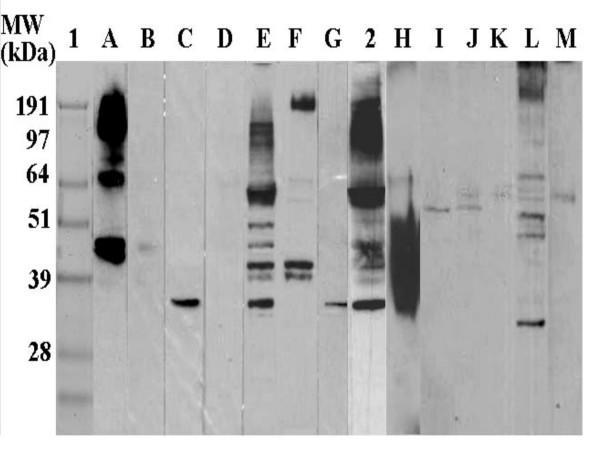
Immunoprint analysis of *B. bassiana *extracts (40 μg crude extract/strip) probed with individual sera. Lane 1) 5 μg protein standards, 2) pooled serum mix-II (patients displaying mould allergies). Lanes A)–G) membrane strips treated with individual sera, Lanes H)–M) membrane strips probed with individuals having had occupational exposure to *B. bassiana *and other fungi (see intra-dermal skin test results for individuals J–M, Table 2).

**Figure 3:**
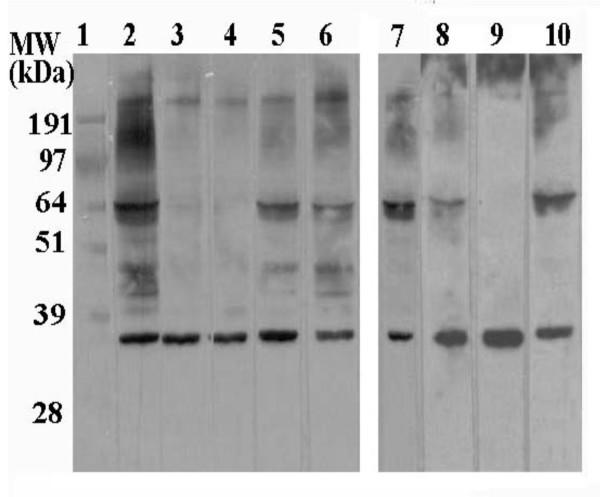
IgE immunoblot inhibition with fungi. *B. bassiana *protein strips (40 μg crude extract) were blocked and incubated with mix containing (500 μl) pooled sera (mix-II) and 2) no additions, 3) 40 μg *Alternaria alternata *crude extract, 4) 400 μg *Alternaria alternata*, 5) 40 μg *Aspergillus fumigatus*, 6) 400 μg *Aspergillus fumigatus*, 7) 400 μg *Cladosporium herbarum*, 8) 400 μg *Candida albicans*, 9) 400 μg *Epicoccum purpurascens*, and 10) 400 μg *Penicillium notatum *protein.

In order to determine the variation and distribution of serum IgEs reactive to *B. bassiana *extracts, individual sera from patients displaying mould allergies (Fig. 2, lanes A–G) as well as random sera from the general population (Fig. 2, lanes H–M) were used as probes for Western blots (Fig. 2). The reactivity of pooled sera from patients A–G (termed serum mix-II) is also shown (Fig. 2, lane 2). Skin prick test results for patients A–G are shown for comparative purposes (Table 1) and represent the clinically determined reactivity of each patient to extracts of the tested fungal species. Patients (A–G) were selected based skin prick reactivity to at least 4 different fungi with scores of 2 or greater. Identical concentrations of *B. bassiana *extract (40 μg) were resolved by SDS-PAGE, blotted to PVDF membranes, and the lanes were cut into separate strips. Each strip was treated with a 1:5 dilution of each respective serum as described in the Materials and Methods (Fig. 3, lane 2 is the sera pool). A total of 16 individual sera were tested, with the sera from three patients displaying no IgEs reactive to proteins present in the *B. bassiana *extracts. The results for the remaining 13 sera are shown in Fig. 2. The data show a large individual variation in serum IgEs capable of binding epitopes present in *B. bassiana *extracts, both in terms of banding distribution and the intensity of the reaction. No correlation was observed between measurements of total IgE and the observed binding to *B. bassiana *allergens. Some patients displayed strong reactions to multiple bands, whereas others to a more limited set of epitopes. Distinct strongly reactive bands ranging from 40 kDa to approximately 200 kDa could be seen in sera A, E, and to a lesser extent L. A strongly reactive 35 kDa band was visible in sera C, G, E, and L. Several sera displayed IgEs that bound to only a limited set of 2–3 allergens (C, F, G, weak bands in B, I, J, K, and M). Blots probed with one serum (H) resulted in a large smear ranging from ~30 kDa to 55 kDa. The reason for the observed smear is unknown and efforts to distinguish separate bands by manipulating the concentrations of either sera or extract were unsuccessful. A number of bands (based upon molecular mass) appeared to be common to several sera including proteins of approximately 35, 42–48, and 60 kDa. A number of allergens of high molecular weight (~100–200 kDa) were also visible; however the resolution in this range on the Western blots is poor.

### Intradermal Skin Testing

A total of ten individuals were tested for allergenic reactivity to *B. bassiana *crude extracts using an intradermal delivery procedure. Data using 1 mg/ml *B. bassiana *crude extract and histamine controls are presented in Table 2. Seven out of the ten individuals (ID #s, J–O, and Q) displayed skin reactivity reactions to the *B. bassiana *extracts (Table 2, also see corresponding Western blot results for individuals J, K, L, and M; Fig. 2). It is interesting to note that 4 (J–M) of 5 individuals (plus S) that have had occupational exposure to *B. bassiana *displayed skin reactivity as well as bands on Western blots. A preliminary correlation was observed between the *B. bassiana*/histamine ration and the *in vitro *reactivity of individual sera in Western blots. Individuals J, K, and M, displayed *B. bassiana*/histamine control ratios <1, also showed weak bands in Western blots (Fig. 2), whereas individual L who had a *B. bassiana*/histamine ratio = 1.65, reacted against numerous epitopes in the extract and with a higher intensity.

**Table 2:** Intradermal skin test results using *B. bassiana *extract

Patient ID	Histamine control^1 ^(0.1 mg/ml)		*B. bassiana *Extract (1 mg/ml)		*B. bassiana*/Histamine
	
	Induration^2^	Erythema^2^	Induration^2^	Erythema^2^	Induration ratio^3^
J^4,5^	7 × 6	12 × 16	8 × 8	12 × 13	0.65
K^4,5^	20 × 15	55 × 50	13 × 12	14 × 13	0.52
L^4,5^	11 × 10	16 × 33	13 × 14	26 × 28	1.65
M^4,5^	15 × 16	36 × 44	10 × 12	10 × 12	0.30
N	16 × 14	38 × 58	10 × 11	21 × 17	0.49
O	21 × 16	39 × 59	9 × 8	18 × 21	0.21
P	15 × 17	44 × 45	5 × 4	5 × 4	0.08
Q	15 × 14	36 × 38	9 × 12	10 × 13	0.51
R	15 × 15	55 × 38	4 × 4	11 × 13	0.07
S^4^	20 × 19	38 × 43	4 × 4	4 × 4	0.04

^1^In all instances saline control injections produced indurations of 3–4 mm × 3–4 mm.

^2^Values recorded in mm × mm.

^3^Induration ratio expressed as *B. bassiana *reaction area (mm^2^)/histamine reaction area (mm^2^).

^4^Individual with occupational exposure to *Beauveria bassiana*.

^5^See Western Blot results for individual, Fig. 2.

### Cross-reactivity among different fungi

In order to determine the extent of cross-reactivity of *B. bassiana *allergens with other fungi, immunoblot inhibition experiments were performed. Identical concentrations of *B. bassiana *crude extract (40 μg) were resolved by SDS-PAGE, blotted to PVDF membranes, and lanes were cut into separate strips. Each strip was treated with a 1:5 dilution pooled sera (serum mix-II) as the primary antibody supplemented with concentrations of fungal crude extracts as described in the Materials and Methods. Fig. 3 shows Western blots in which the binding of human IgEs to allergens present in *B. bassiana *extracts were competed with: excess crude extracts from *Alternaria alternata *(Fig 3, lanes 3, 4), *Aspergillus fumigatus *(lanes 5, 6), *Cladosporium herbarum *(lanes 7)*, Epicoccum purpurascens *(Lane 8)*, Penicillium notatum *(lane 9)*, *and *Candida albicans *(lane 10). There was complete loss of all signals using 2-fold excess *B. bassiana *extract as the competitor (data not shown). These data indicate that while *Beauveria *possess many epitopes in common with several other fungi, notably *Alternaria *and *Penicillium*, a 35-kDa major reactive band was not inhibited by any extract tested.

## Discussion

Although it is well known that fungi are important triggers of respiratory allergies, the potential allergenicity of entomopathogenic fungi used in biocontrol has largely been untested. Aerobiological surveys of conidial fungi and skin sensitivity tests to fungal extracts performed in the 1980s in the Netherlands revealed that although *Beauveria *could barely be detected in airborne samples, and represented less than 0.1% of the airborne fungal "flora", the incidence of allergic skin test reaction to *Beauveria *was the highest of all fungal species tested [10,23,24]. In rural areas, the use of fungi in agricultural pest management practices can greatly increase the potential for human exposure to these agents. Likewise, in urban settings, the commercialization of fungal products for household use may potentiate a much wider problem since indoor air concentrations of the moulds can greatly increase. For these reasons, an examination of the allergenic potential of *Beauveria bassiana *is imperative.

The present study demonstrated the allergenic potential of *B. bassiana *directly by intradermal skin testing of individuals and *in vitro *by revealing the presence of serum IgEs capable of binding allergens present in fungal crude extracts. Over 20 different IgE binding proteins were observed using Western blots probed with sera from patients displaying mould allergies. Results using individual sera revealed a wide variation in IgE-binding proteins between sera, although several common bands, including a protein with an apparent molecular mass of 35 kDa were visible among the sera of several patients.

Our *in vitro *observations were confirmed by intradermal skin testing on individuals using *B. bassiana *extracts. While the testing sample population was small, these results indicated that our extracts were able to elicit allergic reactions in individuals, including some that have had occupational exposure to the fungus. Concentrations of ~1 mg/ml of *B. bassiana *extracts were required to elicit indurations equivalent to 0.1 mg/ml histamine in most individuals, indicating the possibility of potent allergens in the *Beauveria *extract. Interestingly, not all individuals specifically exposed to *B. bassiana *displayed allergic reactions and individuals J, K, and M (who did display mild allergic reactions, Table 2) did not react to the 35 KDa protein based upon Western blotting results (Fig. 2). We do not, however, have any quantifiable index of exposure for the individuals in our sample and any interpretations should be made with some caution.

Numerous studies have revealed the presence of cross-reactive proteins among fungal species between genera [15,20-22,25-27]. In our experiments, (excess) crude extract from a test organism was added during the primary antibody (human sera) incubation. Common or shared epitopes between *B. bassiana *and the test fungus would result in a loss of signal due to competition for reactive IgEs. However, IgEs reactive to *Beauveria-specific *allergens would not be affected, resulting in no change in the corresponding reactive bands on a Western blot. Loss of a signal would indicate that a homolog or shared epitope (IgE-reactive) exists between the two fungal species, implying that primary sensitization by one organism can result in an allergic reaction when exposed to the homologous allergen of another organism. Competitive immunoblot inhibition experiments revealed significant epitope homology between *B. bassiana *and several clinically important fungi responsible for IgE-mediated allergic reactions in atopic individuals. Thus, an allergic reaction to *Beauveria *exposure may arise in patients sensitized to other fungi. Extracts from *A. alternata *and *E. purpurascens *almost completely competed with allergens present in the *B. bassiana *extract with the notable exception of the ~35 kDa allergen. Competition experiments using *A. fumigatus*, *C. herbarum*, *C. albicans*, and *P. notatum *extracts also indicated the presence of many shared epitopes, although distinct (non-competed) IgE-binding *B. bassiana *proteins of 35 kDa, 64 kDa, and >200 kDa molecular mass were detectable. These proteins, particularly the 35 kDa allergens may represent *B. bassiana *specific allergens. Experiments are underway to characterize the 35 kDa allergen, which may lead to a diagnostic assay for *B. bassiana *sensitization. Finally, our analysis of potential *B. bassiana *allergens was limited to cell extracts grown under specific conditions and did not include the culture filtrate. Extracellular proteases, an important class of fungal proteins that can elicit allergenic reactions, have been characterized from a number of fungal species [28-31], and are likely to be present in *B. bassiana*. A careful examination of culture growth conditions is also warranted in order to provide a standardized reagent for testing purposes.

## Conclusions

Although *Beauveria *holds promise as an arthropod biological control agent, there have been few reports on the allergenic potential of these organisms. Identification of *B. bassiana *specific allergens can lead diagnostic methods for determining sensitization to this organism and may provide a rational basis for allergen attenuation in order to yield safer biocontrol products. The observed cross-reactivity among proteins of *B. bassiana *and the fungi tested, highlight the importance of considering the possibility that multiple fungal sensitivity can occur due to exposure to a single fungus. Further testing should be performed to determine the scope, severity, and range of allergenic reactions to *B. bassiana*.

## Competing Interests

The author(s) declare that they have no competing interests.

## Authors' contributions

GSW carried out the immunoassays and other *in vitro *experiments. SWH performed the clinical experiments and participated in the design of the study. NOK conceived of the study, and participated in its design and coordination, and drafted the manuscript.
